# Fluorescence-Based Absent Allele-Specific Amplification (FAASA) for High-Throughput Detection of Absent Alleles

**DOI:** 10.21769/BioProtoc.5633

**Published:** 2026-03-20

**Authors:** Katherine L. D. Running, Sudeshi Seneviratne, Zengcui Zhang, Gurminder Singh, Jason D. Fiedler, Justin D. Faris

**Affiliations:** 1 Department of Plant Sciences, North Dakota State University, Fargo, ND, USA; 2 USDA-Agricultural Research Service, Cereal Crops Improvement Research Unit, Edward T. Schafer Agricultural Research Center, Fargo, ND, USA

**Keywords:** Genotyping, KASP, Marker-assisted selection, PACE, Presence/absence variation, Wheat

## Abstract

In wheat and other crops, some genes display presence/absence variation, and it is occasionally beneficial to select for the absent allele to remove a functional gene. However, current high-throughput genotyping methods used to detect the absence of genes tend to be inconsistent and inconclusive. Kompetitive allele-specific PCR (KASP) and PCR allele competitive extension (PACE) are two well-established methods for allele-specific polymerase chain reaction (AS-PCR) assays, each using fluorescence resonance energy transfer (FRET) to generate a signal for each allele, typically targeting biallelic single-nucleotide polymorphisms. KASP and PACE methods are more difficult to apply to alleles with presence/absence variation because the lack of amplification of the absent allele is indistinguishable from a failed PCR. Here, we present a multiplex fluorescence-based absent allele–specific amplification (FAASA) method using the PACE marker system (compatible with KASP markers) to detect the absence of one particular or all alleles of a target sequence using a primer mix consisting of one target-specific primer pair (TSP) and a second primer set specific to a highly conserved endogenous gene known as a core gene–specific primer pair (CGSP). The forward primer of each pair is tagged with a 5′ terminal tail complementary to dye-labeled oligonucleotides in commercially available FRET cassettes. Lines that amplify only the core gene do not carry the target, while lines that amplify both the core gene and the target carry alleles of both the core gene and the target. The inclusion of the CGSPs allows researchers to confidently distinguish lines with absent alleles of the target from lines with failed PCR reactions, which can happen due to various reasons, including inadequate DNA quality or quantity.

Key features

• A robust, affordable, high-throughput genotyping method for genes or other target sequences with presence/absence variation.

• FAASA markers can be easily incorporated into established marker-assisted selection programs in labs using KASP and/or PACE markers.

• FAASA markers can also be used for other genotyping applications like GWAS, QTL, or bi-parental mapping studies.

• Easily adaptable to different targets and species of interest.

## Graphical overview


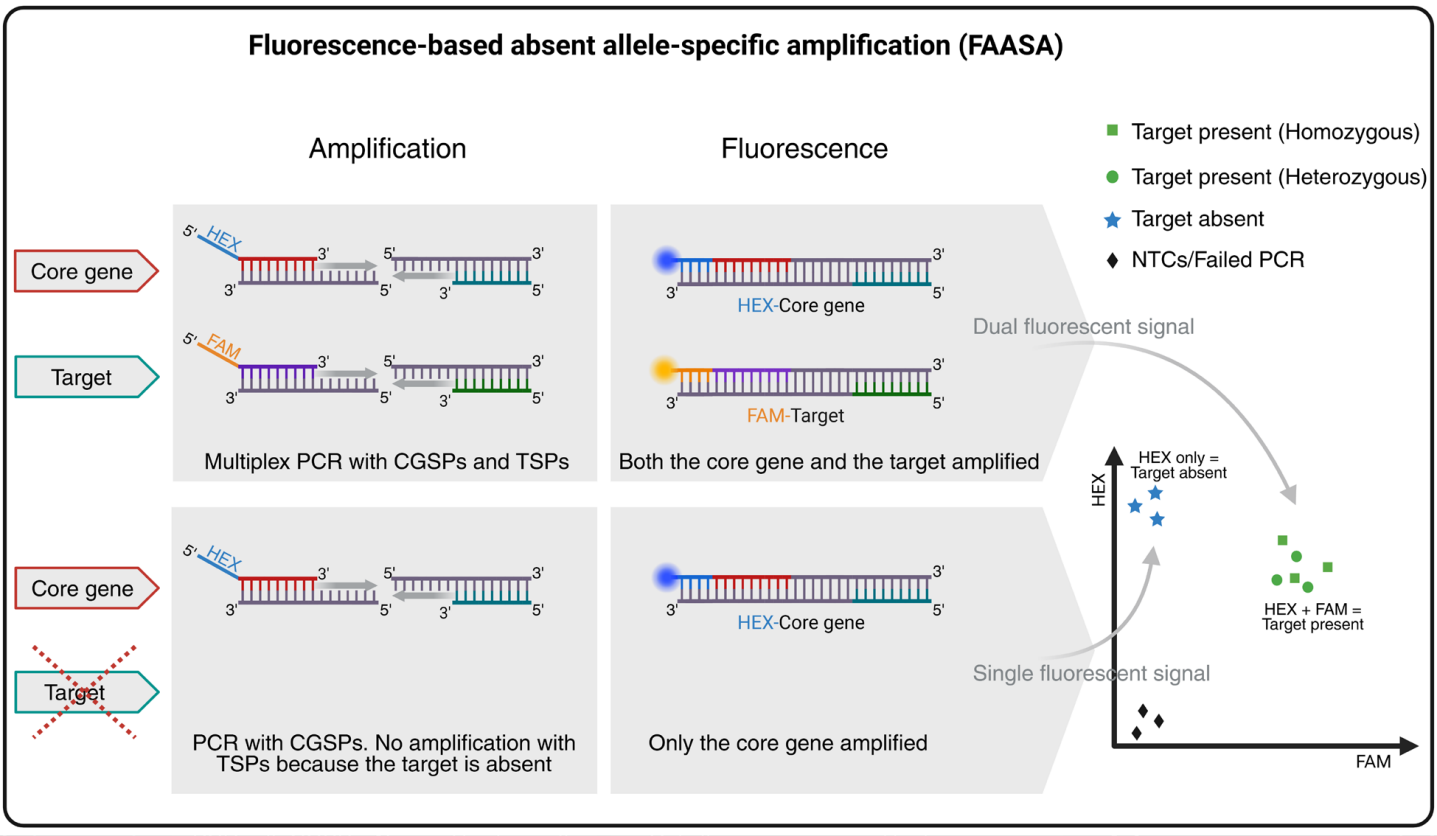


**Overview of the fluorescence-based absent allele–specific amplification (FAASA) methodology.** Created with BioRender.com.

## Background

In wheat, there are certain genes, such as disease susceptibility genes, that need to be eliminated to develop favorable varieties. However, developing a diagnostic high-throughput marker for marker-assisted selection of genes that show presence/absence variation is challenging. The absence of the target gene results in no amplification during PCR, which makes it impossible to distinguish a sample with a truly absent allele from one with a failed PCR, which can occur due to technical issues. Here, we present a high-throughput marker system named fluorescence-based absent allele–specific amplification (FAASA) to confidently detect absent alleles of any target of interest (gene, a particular allele of a gene, promoter, indel, etc.).

This approach is based on multiplex PCR, a technique first described by Chamberlain et al. in 1988 [1] that has since been used in various assays [2–4]. In general, multiplex PCR refers to the amplification of multiple amplicons simultaneously during PCR using more than two primers in the same reaction mixture. It is commonly used in allele-specific PCR assays such as kompetitive allele-specific PCR (KASP) and PCR allele competitive extension (PACE) genotyping systems [5], where two amplicons are amplified based on a single-nucleotide polymorphism (SNP) between two alleles of the same target sequence. Adaptations of the original KASP/PACE systems to amplify two target regions, rather than SNP variations, have also been reported [6–8]. In FAASA, we utilize the PACE system to simultaneously amplify two distinct genomic regions (target and core gene) to enable high-throughput detection of absent alleles. Here, a total of four primers are used in a multiplex PCR: one pair of target-specific primers (TSPs) and one pair of core gene–specific primers (CGSPs). The forward primers of both TSPs and CGSPs are tagged with FAM and HEX (fluorescein amidite and hexachlorofluorescein) fluorescent tails, respectively. The core gene is expected to be present in all biological samples. As a result, the CGSPs will consistently amplify a fragment of the core gene, producing a HEX fluorescent signal. This signal serves as an internal control, indicating that the PCR reaction was successful. Simultaneously, the TSPs will amplify a fragment of the target, generating a FAM signal if the target is present in the sample. As a result, samples containing the target will produce both FAM and HEX signals. These overlapping signals create a combined fluorescence pattern that typically appears as a cluster in the middle of the endpoint fluorescence scatter plot, resembling a heterozygous cluster. However, this is not due to actual heterozygosity but rather to the simultaneous detection of both amplified core gene and target products. If the target is absent from a sample, only the core gene will be amplified, resulting in a HEX-only signal. These samples will form a separate cluster corresponding to the absence of the target. Samples with failed PCR reactions and non-template controls (NTCs) will show no amplification for either of the genes and will cluster in the bottom-left corner of the scatter plot due to the absence of both signals. While we report the use of FAM for the target and HEX for the core gene, any two compatible fluorescent dyes may be used, and their assignment may also be switched if preferred.

While this method can reliably detect the absence of a target, it is important to note that it does not distinguish between homozygous and heterozygous states when the target is present. However, FAASA can confidently and efficiently differentiate truly absent alleles from PCR failure, which can be particularly valuable for plant breeders during large-scale marker-assisted selections, often allowing the bypass of exhaustive phenotypic screens. While FAASA markers can be designed to detect the absence of a particular allele or all alleles of a target sequence, they cannot account for accrued mutations within the target that are not targeted by the TSPs that potentially render the target nonfunctional. Thus, if there are multiple segregating mutations in a target that produce the same phenotype, multiple FAASA markers may be needed to adequately detect all samples with alleles producing that phenotype. This method can be applied more broadly to detect any target in a genome, genic or non-genic, as long as primers can be designed to specifically amplify the target of interest. Therefore, these markers can also be used in molecular mapping projects. While we present an example in this protocol using a FAASA marker that amplifies wheat target and core genes, FAASA markers may be used in other species and scenarios as well.

## Materials and reagents

**Biological materials**

1. 45 ng of dsDNA or 90 ng of total DNA per biological sample per replicate; include at least one control sample with the target as a positive control and a sample without the target as a negative control. Appropriate controls are described in more detail in section A.

*Note: This quantity of DNA is specific to wheat, which has a very large genome. If applying this method to a species with a smaller genome, less DNA may be needed, as the relative concentration of the target and core genes will be higher in a smaller genome.*

**Reagents**

1. ddH_2_O

2. One pair of target-specific primers (TSPs) (see section A)

a. 100 μM FAM tagged TSP (5′ FAM terminal tail “GAAGGTGACCAAGTTCATGCT”)

b. 100 μM reverse TSP

c. The following target-specific primers for the FAASA marker *fcp1069* were used in this protocol as an example: Tsc1-1Ka-Null-Fam: GAAGGTGACCAAGTTCATGCTATAAACGAAAGATACTTGTTTTGCTC, Tsc1-1Ka-Null-Rev: ATTATTATGACAGAAATTGCAACAACA

3. One pair of core gene–specific primers (CGSPs) (see section A)

a. 100 μM HEX tagged CGSP (5′ HEX terminal tail “GAAGGTCGGAGTCAACGGATT”)

b. 100 μM reverse CGSP

c. The following core gene–specific primers were used for the marker *fcp1069* in this protocol: Q_KB.1-Hex: GAAGGTCGGAGTCAACGGATTGCTAATTAAACGTCCACAGCAT, Q_KR.1-Rev: GACACTAATTAGTAGTAGATGTGACAG

*Note: The sequences provided for the TSPs and CGSPs are for marker fcp1069, targeting the wheat genes* Tsc1 *and* Q *gene, respectively. Marker fcp1069 can be used to detect the absence of the tan spot susceptibility gene* Tsc1 *in wheat. The CGSPs can be used in conjunction with other TSPs to create FAASA markers to detect the absence of other targets in wheat.*

4. 2× PACE genotyping master mix with standard ROX concentration (3CR Bioscience, catalog numbers: 001-0001, 001-0002, 001-0003, or 001-0004)

*Note: Fluorescent probes in the master mix are light sensitive. To minimize exposure to light and prevent degradation from repeated freeze-thaw cycles, freeze small aliquots at -20 °C. Thaw 2× PACE genotyping master mix in a drawer or under cover. There are commercially produced light-blocking microcentrifuge tubes that may be a better option if light exposure cannot be adequately controlled. Use the FAASA master mix immediately.*

5. 70% (v/v) ethanol

**Solutions**

1. FAASA primer mix (see Recipes)

2. FAASA master mix (see Recipes)

**Recipes**

**1. FAASA primer mix**

**Table:** 

Reagent	Final concentration	Quantity or volume
100 μM FAM tagged TSP (Tsc1-1Ka-Null-Fam)	12 μM	12 μL
100 μM reverse TSP (Tsc1-1Ka-Null-Rev)	30 μM	30 μL
100 μM HEX tagged CGSP (Q_KB.1-Hex)	12 μM	12 μL
100 μM reverse CGSP (Q_KR.1-Rev)	30 μM	30 μL
ddH_2_O		16 μL
Total	73×	100 μL

*Note: The primers in parentheses are the primers for marker fcp1069 used as an example in this protocol. Other primers may be used. Store at -20 °C and bring to room temperature before use.*

**2. FAASA master mix**

**Table:** 

Reagent	Final concentration	Volume per reaction	Volume for 16 reactions	Volume for 96 reactions	Volume for 384 reactions
FAASA primer mix	1×	0.055 μL	1.375 μL	6.05 μL	23.10 μL
2× PACE genotyping master mix	1×	2 μL	50 μL	220 μL	840 μL
ddH_2_O		2 μL	50 μL	220 μL	840 μL
Total		4.055 μL	101.375 μL	446.05 μL	1,703.1 μL
Volume per well in 8 wells			11 μL (2.75 reactions)	54 μL (13.5 reactions)	210 μL (52.5 reactions)

*Notes:*

*1. Volumes for 16, 96, and 384 reactions include extra to account for volume lost during pipetting due to the saponaceous quality of 2× PACE genotyping master mix.*

*2. It is very difficult to see if there is liquid already pipetted into a well because the wells are opaque. We recommend dividing the master mix into 8 wells in a column on a PCR plate or strip and using a multichannel pipette to distribute the FAASA master mix to the PCR plate to reduce the chance of pipetting errors. The volume to put in each well is provided in the recipe.*

**Laboratory supplies**

1. 20, 250, and 1,000 μL pipette tips (Rainin, catalog numbers: 17005872, 17005874, and 17007089, or similar)

2. (Optional) Filtered 20, 200, and 1,000 μL pipette tips (Rainin, catalog numbers: 30389227, 30389239, and 30389212, or similar)

3. 2, 20, and 1000 μL pipette (Rainin, catalog numbers: 17014393, 17014392, 17014382, or similar)

4. 10 μL multichannel pipette (Rainin, catalog number: 17013802)

5. 96-well PCR plate (BrandTech, catalog number: 781368, or similar)

6. (Optional) 8-well PCR strip tubes (BrandTech, catalog number: 781326, or similar)

7. 384-well PCR plate (Bio-Rad, catalog number: HSP3805, or similar)

8. TempPlate RT optical film (USA Scientific, catalog number: 2978-2100)

9. (Optional) AxySeal sealing film (Axygen, catalog number: PCR-SP, or similar)

10. MicroAmp^TM^ adhesive film applicator (Thermo Fisher Scientific, catalog number: 4333183, or similar)

11. 1.7 or 2 mL microcentrifuge tubes, depending on master mix size (Fisherbrand, catalog numbers: 02-681-331 or 02-681-332, or similar)

## Equipment

1. Real-time PCR machine or thermocycler and fluorescent plate reader (Bio-Rad CFX Opus 384 Real-Time PCR System, model: 12011452)

2. Spectrophotometer (Nanodrop, model: ND 8000)

3. Vortex (Fisherbrand, catalog number: 02-215-414)

4. Plate mixer (Eppendorf MixMate, catalog number: 5353000529)

5. Centrifuge with plate adapters (Eppendorf, model: Centrifuge 5810 R)

6. Incubator (Fisherbrand, catalog number: 15-103-0513, or similar)

## Software and datasets

1. CFX Maestro (license required, comes with Bio-Rad CFX Opus 384 Real-Time PCR System), Bio-Rad, versions 1.1–2.3, or similar clustering software

2. (Optional) Primer3 (available online for free at https://www.ncbi.nlm.nih.gov/tools/primer-blast/)

## Procedure

**A. Design primers**

1. Design four primers in total: one pair targeting a specific region of the target sequence (TSPs) and another pair targeting a conserved region of the core gene (CGSPs). A primer designing tool like Primer3 may be helpful to ensure your primers meet the guidelines below. If working with an A subgenome-containing *Triticum* species (*Q* gene-containing), the provided CGSPs listed in Reagents may be used.

2. Follow these guidelines when designing the primers:

a. Design CGSPs to target a single copy gene that is present in all or nearly all individuals of the species of interest. Multi-copy genes will skew amplification efficiencies.

b. Design primers that generate short amplicons. We recommend having 5–100 bp between the 3′ ends of the forward and reverse primers.

c. The optimal melting temperature (T_m_) of each primer is 57–60 °C. For best results, the T_m_ of the TSPs and CGSPs should be within 1–2 °C, excluding the fluorescent tails.

d. Each primer should be 18–30 bp in length, excluding the fluorescent tails.

e. The GC content should be between 40% and 60%, excluding the fluorescent tails.

f. Avoid having more than three consecutive G or C bases within the last 5 bases at the 3′ end.

g. Avoid secondary structures such as hairpins, self-dimers, and cross-dimers.

h. Avoid repetitive sequences and long stretches of a single nucleotide (homopolymers).

**Critical**: Primers (CGSPs and TSPs) need to be specific to the DNA sequence they are amplifying. This is especially important at the 3′ end of the primer. While it is ideal for both forward and reverse primers to be specific, it is essential that at least one primer has high specificity at its 3′ end (i.e., has multiple SNPs or indels distinguishing the primer sequence of the target or core gene from similar DNA sequences).

3. Add the FAM fluorescent tail (5′ GAAGGTGACCAAGTTCATGCT 3′) to the 5′ end of the forward TSP.

4. Add the HEX fluorescent tail (5′ GAAGGTCGGAGTCAACGGATT 3′) to the 5′ end of the forward CGSP.

5. Order synthesized primer sequences from a preferred DNA oligo synthesizing company.

**B. Marker test**

Before evaluating a large set of samples with the marker, it is important to ensure that the marker assay is specific, polymorphic, and generates clearly distinguishable clusters by evaluating a subset of samples with known allelic states for the target.

1. Prepare the FAASA primer mix (see Recipes).

2. Select representative controls, including known positive, negative, and NTCs as described in section C. Selected controls should reflect the expected variation in the sample set.

3. Prepare 2–4 replicates of each control to assess accuracy and reproducibility.

4. Perform the PCR as described in sections D and E and analyze the resulting scatter plot as described in section F.

a. Ensure tight clustering within each control type and its replicates.

b. Ensure clear separation between the clusters.

c. Confirm there are no unintended amplifications.

5. Run additional cycles as needed. The standard PCR run includes 40 cycles, and additional cycles may be added in 3-cycle increments (i.e., 43, 46, and 49 cycles) to improve signal intensity and cluster definition. To further evaluate marker performance, we recommend assessing scatter plots generated with different numbers of cycles.

a. An ideal marker will produce clean clusters at all points (0, 3, 6, and 9 additional cycles).

b. Markers that give consistent clustering with 3 and 6 additional cycles are still acceptable.

c. If more than 9 additional cycles are required to obtain a clear signal, the marker is not recommended, as it may lack reproducibility across different labs.

d. If cluster merging or loss of resolution after 3–6 extra cycles is observed, proceed with caution, as this may indicate over-amplification or nonspecific signal accumulation.

6. If the marker test yields clear, consistent, and well-separated clusters, the marker can be confidently used across the full sample set. The target should amplify in the positive control but not the negative control. The core gene should amplify in both the positive and negative controls. No amplification should be observed in the NTCs. When evaluating the full set, we recommend including the controls on every plate to check for consistency and ensure the assay is working properly.

**C. Prepare samples**

1. Select appropriate controls to confirm that the marker reliably and specifically amplifies the target.

a. Positive control: something known to possess the target and core sequence. A positive control could be a differential line, a line from which the target has been sequenced, or a sequenced accession confirmed to have the target in its genome assembly.

b. Negative control: something known to lack the target sequence but contain the core gene. Negative controls should be genetically similar to the test samples to confirm that the primers (TSPs) do not amplify a nonspecific target. It could be an accession with the absent allele of the target, a deletion line (chromosome or segment), or a substitution line. For allopolyploids, it is important that the specificity of the marker be evaluated for all subgenomes present in the test samples; thus, the controls must have all of the subgenomes present in the test samples. For example, if test samples are hexaploid wheat (subgenomes A, B, and D), hexaploid controls should be used. Tetraploid (subgenomes A and B) or diploid controls should not be used to test the specificity of a marker in hexaploid test samples, as it would prohibit the ability to confirm target specificity in all subgenomes present in the test samples.

*Note: For example, if testing the specificity of marker fcp1069, which targets* Tsc1 *on chromosome 1A of wheat, it would be appropriate to use one of the following as a negative control: 1) a tan spot differential line that has the absent allele of* Tsc1 *(e.g., Glenlea, Salamouni, or 6B662) verified by alternative genotyping methods, 2) a sequenced wheat accession with the absent allele of* Tsc1 *(e.g., Jagger, Kronos, Svevo, Lancer, Stanley), or 3) a chromosome substitution line missing chromosome 1A (e.g., Chinese Spring nulli-1A tetra 1B or Chinese Spring nulli-1A tetra 1D).*

c. Non-template control (NTC): Include an equal volume of PCR-grade ddH_2_O in place of template DNA to serve as an NTC. NTCs help detect potential contamination or primer-dimer formation and provide a baseline for the background signal. They typically appear in the bottom-left corner of the endpoint fluorescence scatter plot, aiding in distinguishing failed amplifications and enhancing cluster resolution.

2. Dilute samples to 25–35 ng/μL (if measuring ssDNA and dsDNA) or 12–17 ng/μL (if measuring dsDNA). See General note 3 for an alternative method omitting the DNA drying step.

**Critical**: To generate clear clusters, it is critical that the DNA concentrations of samples are within a narrow range. The more divergent the concentration of the samples, the wider the clusters will be and the harder it will be to distinguish alleles.

**Figure 1.**
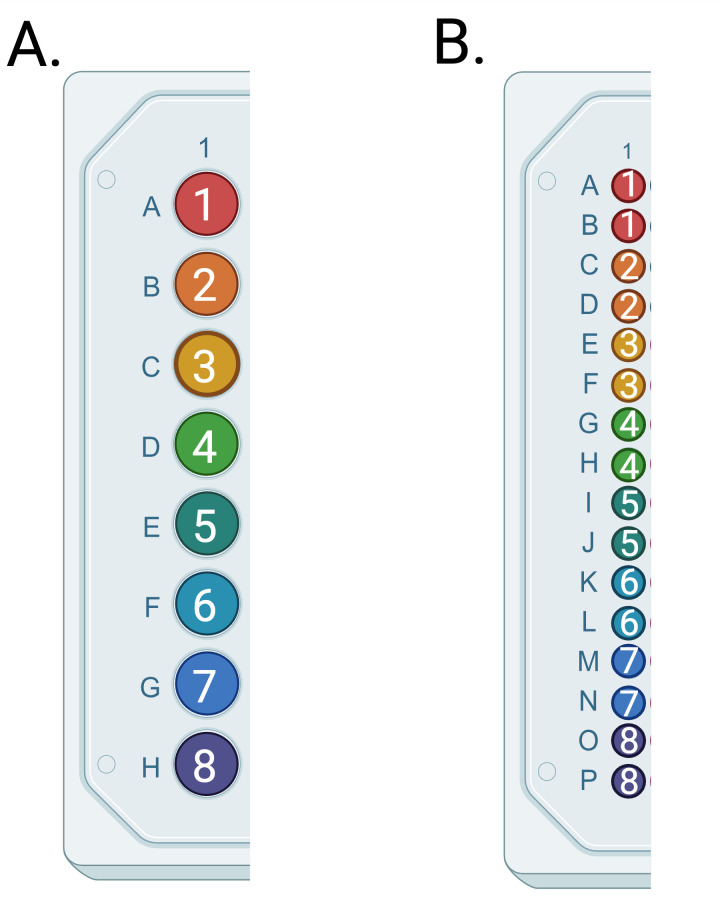
Example plate layouts. (A) Samples 1–8 on the 96-well dilution plate. (B) Samples 1–8 in duplicate on the 384-well PCR plate.

3. Pipette 3 μL of each diluted sample into the bottom of each well of a 384-well plate. Do not touch the pipette tip to the sides of the PCR wells. If it is critical that each sample has an allelic call, samples may be replicated on the 384-well plate. One column of an example dilution plate (96-well) and a 384-well reaction plate are shown in Figure 1, allowing for the use of a multichannel pipette and including each sample twice on the 384-well plate.

3. Cover the plates with an adhesive sealing film and centrifuge at ≥300× *g* for 10 s to ensure DNA is at the bottom of the well.

*Note: Because this sealing film is being used temporarily and will not be the one used for PCR, this sealing film does not need to be optically clear; less expensive sealing films may be used for the preparation of samples (e.g., Axygen AxySeal sealing film).*

4. Remove the sealing film and place the plate in an oven at 65 °C for 1–1.5 h to dry the samples. See General Note 2 for alternative drying temperatures.

5. After the drying step, allow the plate to cool at room temperature for 15–20 min before proceeding with PCR to prevent evaporation of the master mix due to residual heat.

**Pause point**: Alternatively, dried plates may be covered and stored at room temperature for later use.

**D. PCR setup**

1. Keep the laboratory lights dim during PCR setup, if possible, as the 2× PACE genotyping master mix is light sensitive. Always thaw the 2× PACE genotyping master mix in the dark, such as inside a drawer or a covered box.

2. Prepare the FAASA master mix in a microcentrifuge tube using ddH_2_O, FAASA primer mix, and 2× PACE genotyping master mix, following the volumes outlined in Recipe 2. Vortex the FAASA master mix thoroughly.

3. For efficient pipetting, divide the mix into 8-well strip tubes or into a single column of a 96-well plate. Refer to the “Volume per well in 8 wells” row in Recipe 2 for the appropriate volume per well in the strip tubes.

4. Dispense 4 μL of FAASA master mix into each well of the previously prepared 384-well plate. If the template DNA does not contact the side of the PCR well, the 4 μL of FAASA master mix can be dispensed on the top side of the well, and the same set of tips can be reused for the whole plate. Seal the plate securely with a new optically clear adhesive sealing film, ensuring that all edges and corners are fully sealed.

**Critical**: Avoid touching the surface of the sealing film without gloves. Use an adhesive film applicator to press down and ensure a proper seal.

5. Briefly centrifuge the sealed plate at ≥ 300× *g* for 10 s to settle the contents. Mix thoroughly using a plate mixer at 2,600 rpm for 45 s, then centrifuge at ≥ 300× *g* for 10 s.

6. Make sure the plate is sealed and wipe the surface of the sealing film with 70% ethanol. The plate is now ready to be loaded into the thermocycler or real-time PCR machine.

**E. PCR cycling conditions**

1. Perform the PCR according to the cycling conditions specified in Table 1.

**Table 1.** PCR cycling conditions for FAASA Table 1 is adapted from the PACE^®^ genotyping master mix user guide with a modified constant annealing and extension temperature in step 2 [9]. *For a real-time PCR machine, include step 4 before signal detection. If a separate fluorescent plate reader is used, this step is not required, and the plate can be read after step 3.

Step	Description	Temperature	Time	Number of cycles
1	Initial denaturation and enzyme activation	94 °C	15 min	1
2	Denaturation	94 °C	20 s	10
	Annealing and extension	65 °C	1 min	
3	Denaturation	94 °C	20 s	30
	Annealing and extension	57 °C	1 min	
4*	Pre-read cool down	12 °C	10 s	1

2. Read the plate immediately using the real-time PCR machine or a fluorescent reader to detect the fluorescent signals.

*Note: Configure the instrument to detect signals in all or FAM, HEX, and ROX channels.*

3. Add additional cycles in increments of three until well-separated tight clusters are obtained, following the conditions outlined in Table 2. Read the plate after each 3-cycle increment.

**Table 2.** Cycling program for adding extra cycles for FAASA *For a real-time PCR machine, include step 3 before signal detection. If a separate fluorescent plate reader is used, this step is not required, and the plate can be read after step 2.

Step	Description	Temperature	Time	Number of cycles
1	Denaturation	94 °C	20 s	3
2	Annealing and extension	57 °C	1 min	
3*	Pre-read cool down	12 °C	30 s	1

**F. Interpretation of data**

**Figure 2.**
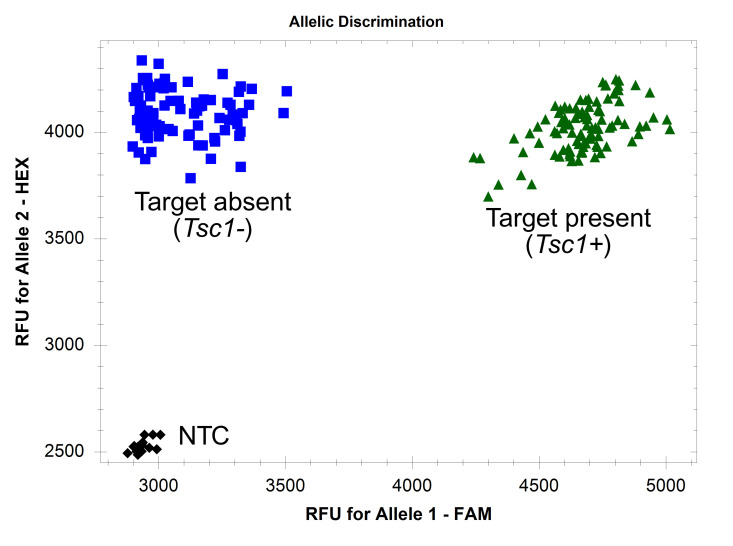
Endpoint fluorescence scatter plots of FAASA marker *fcp1069*. Marker *fcp1069* was run on 94 winter wheat accessions with 49 PCR cycles. Amplification of both the core gene and target is indicated by green triangles, while amplification of only the core gene is indicated by blue squares. Black diamonds indicate no amplification, observed in the NTC.

**Figure 3.**
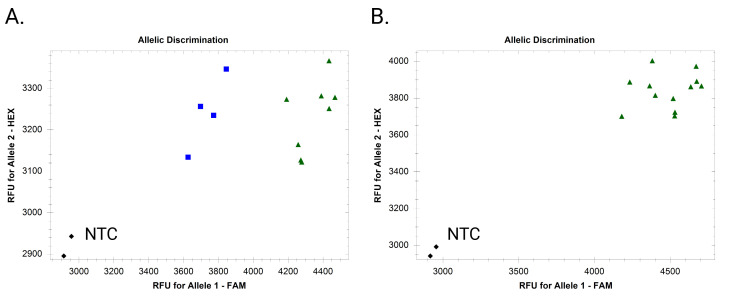
Example endpoint fluorescence scatter plots of a nonspecific fluorescence-based absent allele–specific amplification (FAASA) marker. (A) Inadequate separation of clusters with 0 extra cycles. (B) Cluster merging with three extra cycles.

**Figure 4.**
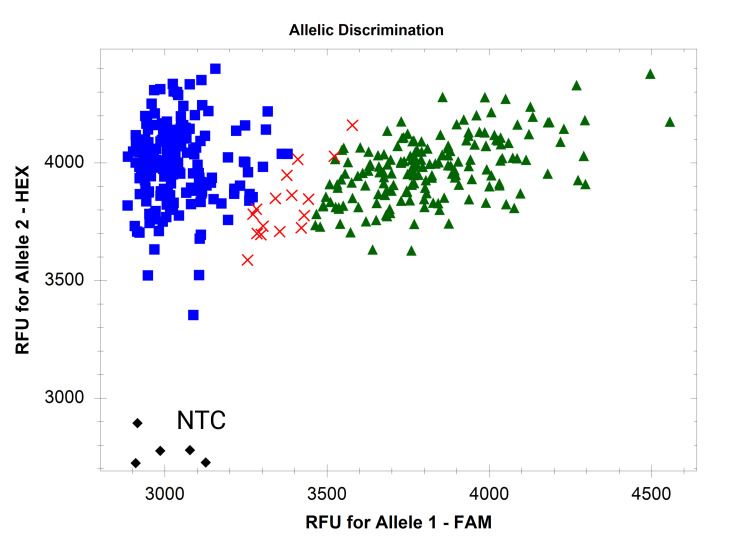
Dispersed clusters due to inconsistent DNA quantity

**Figure 5.**
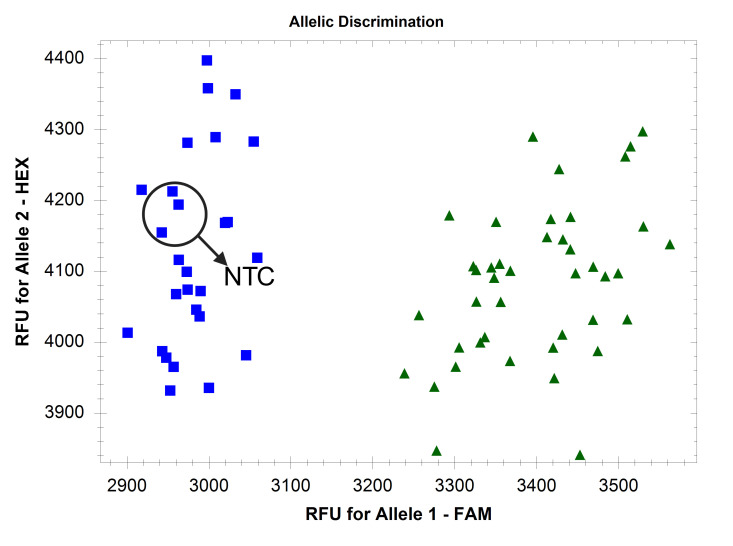
Amplification of allele 2 (core gene) in all samples and non-template control (NTC)

**Figure 6.**
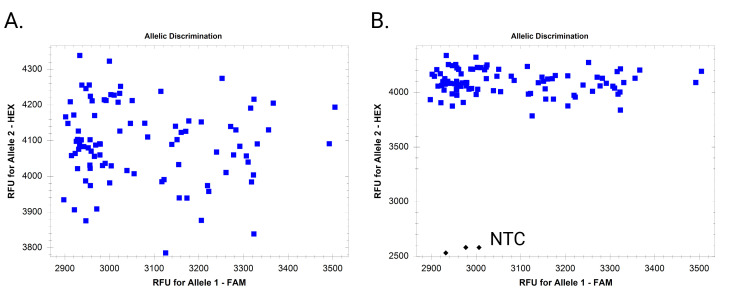
Example endpoint fluorescence scatter plots when only one allele amplifies. (A) Amplification of only one allele without including a non-template control (NTC). (B) Amplification of only one allele with inclusion of NTC.

1. Use cluster analysis software to analyze the HEX and FAM fluorescent signal data. If using a CFX Opus 384 Real-Time PCR System and Bio-Rad CFX Maestro^TM^ 2.3 software, use default settings on the “Allelic discrimination” table. The target is present in samples producing both HEX and FAM fluorescent signals (Figure 2). The target is absent in samples producing only HEX signals. Ensure the negative and positive controls are present in the expected clusters. See Troubleshooting if markers do not perform as expected (Figures 3–6).

*Note: If the CGSPs are FAM tagged and the TSPs are HEX tagged, the target will be absent in samples producing FAM signals.*

## Validation of protocol

This protocol (or parts of it) has been used and validated in the following research article(s):

- Seneviratne et al. [10] Evolution, diversity, and function of the disease susceptibility gene *Snn1* in wheat. *Plant J.* (Figure 9)
- Zhang et al. [11] Protein kinase-major sperm protein (PK-MSP) genes mediate recognition of the fungal necrotrophic effector SnTox3 to cause septoria nodorum blotch in wheat. *MPMI* (Figure 7).

Data presented in this protocol also serve to validate the FAASA methodology. In wheat, the susceptibility gene *Tsc1* interacts with the *Pyrenophora tritici-repentis* necrotrophic effector Ptr ToxC to induce chlorosis and cause the disease tan spot [12]. In the 94 winter wheat samples shown in Figure 3, 100% of the samples predicted to not have a *Tsc1* allele did not produce chlorosis when the accessions were inoculated with a Ptr ToxC-producing isolate of *Pyrenophora tritici-repentis*, indicating the absence of a functional *Tsc1* allele.

## General notes and troubleshooting

**General notes**

1. Although FAASA markers generally perform better when the amplicon is small, larger amplicons may be attempted if specific markers cannot be designed ≤100 bp apart.

2. If an incubator that reaches 65 °C is not available, incubation at 50 °C may suffice.

3. Hydrated DNA may be used in PCR reactions if the DNA cannot be dried. In this case, omit the water in the FAASA master mix, dilute samples to 38–52 ng/μL (if measuring ssDNA and dsDNA) or 18–25 ng/μL (if measuring dsDNA), and pipette 2 μL of DNA into the bottom of each reaction well. Because this is a small volume and it can take some time to fill 384 wells with DNA, we recommend covering columns with tape as the plate is filled to prevent evaporation. Evaporation will result in uneven reaction volumes across the plate. Proceed to section D and add 2 μL of FAASA master mix.

4. If amplification of the target sequence is challenging, consider incorporating a touch-down step into the PCR cycling conditions. Set the annealing extension temperature (step 2 in Table 1) to start at 65 °C and decrease it by 0.8 °C each cycle for the first 10 cycles as described in the PACE^®^ genotyping master mix user guide [11].

*Note: If using a CFX Opus 384 Real-Time PCR System and Bio-Rad CFX Maestro* ^TM^ *2.3 software, relative fluorescence unit (RFU) values below 3,000 typically indicate poor amplification.*

5. The *target present* cluster consists of both homozygous and heterozygous samples, making it impossible to distinguish between them. However, the *target absent* cluster exclusively represents samples that are homozygous for the absent allele.

**Troubleshooting**

**Problem 1**: No clear separation between the clusters, or clusters begin to merge after 3–6 extra cycles.

Possible cause: Primer lacks sufficient specificity.

Solutions:

1. Redesign the primers with improved 3′ end specificity. To do this:

a. Conduct BLAST analysis of the target sequence against multiple whole genomes if available.

b. Select the hits with >90% identity and align these sequences using a multiple sequence alignment tool.

c. Choose a region containing one or more SNPs and design the primer so that its 3′ end aligns with a SNP unique to the target sequence. Having 2–3 SNPs at the 3′ end is ideal to maximize the specificity.

d. Alternatively, designing primers spanning an indel specific to the target sequence would increase specificity.

2. Optimize the PCR cycling conditions. The annealing and extension temperatures (step 3 in Table 1 and step 2 in Table 2) may be increased up to 59–61 °C to enhance the primer annealing specificity.

**Problem 2**: Clusters are present but not tight, or replicates of the same sample do not group closely.

Possible cause: Inconsistent DNA quantity across wells.

Solutions:

1. Ensure that all DNA samples are normalized to a consistent DNA concentration before pipetting into the wells.

2. Ensure that pipetting is accurate and consistent to avoid variation in volume. Check pipette calibration. If reaction volumes are inconsistent, using ROX-normalized RFU values may help.

**Problem 3**: NTCs show amplification.

Possible causes:

1. Primer dimer formation.

2. Contamination of reagents.

Solutions:

1. Redesign the primers with lower complementarity scores. Keep self-complementarity below 8 and 3′ complementarity below 6. Avoid having more than three consecutive G or C bases, especially within the last 5 bases at the 3′ end.

2. Replace reagents with fresh PCR-grade ddH_2_O and a freshly prepared primer mix.

**Problem 4**: Samples appear scattered, and clusters are not visible, although distinct clusters were observed during the initial marker test and on other plates.

Possible cause: Only one type of allele (either the present allele or the absent allele) is present in the selected sample set, or only one set of primers was included in the FAASA primer mix. Note the differences in scatter plot scales compared to the marker test to diagnose.

Solution: Include multiple replicates of control samples (see sections B and C) in addition to test samples on the same plate. In order to observe distinct clusters on a scatter plot, both types of alleles must be present within the same sample set. Confirm if all four primers are present in the FAASA primer mix.
